# Rational strategies for enhancing mAb binding to SARS-CoV-2 variants through CDR diversification and antibody-escape prediction

**DOI:** 10.3389/fimmu.2023.1113175

**Published:** 2023-03-31

**Authors:** Masaud Shah, Ji-Yon Shin, Hyun Goo Woo

**Affiliations:** ^1^ Department of Physiology, Ajou University School of Medicine, Suwon, Republic of Korea; ^2^ Korea Initiative for Fostering University of Research and Innovation (KIURI) Program, Ajou University School of Medicine, Suwon, Republic of Korea; ^3^ Department of Biomedical Science, Graduate School, Ajou University, Suwon, Republic of Korea

**Keywords:** SARS-CoV-2, omicron, Omicron (B.1.1.529), immune escape, antibody, CDR diversification

## Abstract

Since the emergence of SARS-CoV-2, dozens of variants of interest and half a dozen variants of concern (VOCs) have been documented by the World Health Organization. The emergence of these VOCs due to the continuous evolution of the virus is a major concern for COVID-19 therapeutic antibodies and vaccines because they are designed to target prototype/previous strains and lose effectiveness against new VOCs. Therefore, there is a need for time- and cost-effective strategies to estimate the immune escape and redirect therapeutic antibodies against newly emerging variants. Here, we computationally predicted the neutralization escape of the SARS-CoV-2 Delta and Omicron variants against the mutational space of RBD-mAbs interfaces. Leveraging knowledge of the existing RBD-mAb interfaces and mutational space, we fine-tuned and redirected CT-p59 (Regdanvimab) and Etesevimab against the escaped variants through complementarity-determining regions (CDRs) diversification. We identified antibodies against the Omicron lineage BA.1 and BA.2 and Delta variants with comparable or better binding affinities to that of prototype Spike. This suggests that CDRs diversification by hotspot grafting, given an existing insight into the Ag-Abs interface, is an exquisite strategy to redirect antibodies against preselected epitopes and combat the neutralization escape of emerging SARS-CoV-2 variants.

## 1 Introduction

After the emergence of SARS-CoV-2, the virus has been in a relative evolutionary stasis, but as the virus began to evolve by the end of 2020, and new variants continue to emerge. This has been characterized by the identification of variants of concerns (VOCs) and variants of interest (VOIs). These VOCs are characterized as rapidly transmissible, immune evading, and more pathogenic in some cases, likely due to the variable immune profile of the host population as the virus spread across the globe (1). Among the first four VOCs, namely Alpha, Beta, Gamma, and Delta, the Delta variant was expected to pose a challenge to the neutralization of FDA-approved monoclonal antibodies (mAbs) (2) and the effectiveness of FDA-approved vaccines, as the neutralization efficacy of the BNT162b2 vaccinees sera dropped by 5.8-fold and that of Pfizer vaccine dropped by 3-fold (3). More worrisome is that the neutralizability of the single dose AstraZeneca vaccine is completely lost and infection can occur even after two doses of the vaccine (4). Among the four FDA-approved mAbs of Regeneron and AbCellera & Eli Lilly under Emergency Use Authorization (EUA) at that time, Bamlanivimab completely lost its neutralization against the delta variant. A single mutation L452R in the RBD^delta^ located at the RBD-mAb interface was responsible for the loss in neutralizing activity (4), suggesting that one hotspot mutation in the emerging SARS-CoV-2 variants can allow for escape from highly effective mAbs that have undergone through extensive biosafety evaluation and clinical trials. Similarly, the neutralization of the sera from the convalescent donors after vaccination with BNT162b2 fell 7.6-fold for the Mu variant, which was substantially worse than other VOCs (3).

The new VOC, Omicron, and its subvariants contain at least 15 mutations in the RBD region alone (5). Due to the triple mutations H655Y, N679K, and P681H in the furin cleavage site (6), this variant is thus far known to be the fastest spreading variant (7). Moreover, several single mutations in potential epitopes that overlap with the ACE2-binding interface have been reported to escape neutralizing antibodies (nAbs) (8, 9). Particularly, Lys417Asn, Gly446Ser, Leu452Arg, Glu484Ala, and Gln493Arg mutations play a major role in this escape. Based on sufficient data gathered on the neutralization efficacy of mAbs against Omicron, the FDA has amended its approval for the use of two mAbs-based cocktail therapies in COVID-19. Patients can benefit from the use of casirivimab and imdevimab (used as cocktail therapy) and Etesevimab and bamlanivimab (administered together) only if they are exposed to a variant susceptible to these treatments. As Omicron and its sub-variants are fully resistant to individual or cocktail treatment of these mAbs (10).

A recent cohort study involving 936 SARS-CoV-2 convalescent patients found ultra-potent broad-spectrum antibodies as “elite neutralizers” from a subset of the convalescent individuals (11). Where some of these mAbs broadly neutralize SARS-CoV-2 VOCs and Omicron sub-variants, others could be resisted due to single point mutations within Omicron sub-variants (12). While mapping the epitopes of these mAbs in our ongoing study, we found that R40-1C8 (mAb) neutralize Wuhan strain and four sub-variants of Omicron, failed to neutralize BA.4/5 variant due to Phe486Val mutation (unpublished). Supporting this notion, Bebtelovimab that broadly neutralizes all SARS-CoV-2 variants was recently reported to be less effective against BQ.1.1 sub-variant of the Omicron that was recently emerged (13). We found that this loss in neutralization was due to the breakage of crucial salt bridges between Lys444 and electrostatic amino acids in CDRH2 of the antibody due to Lys444Thr mutation in RBD (unpublished). However, antibodies that recognize conserved epitopes on RBD and hold pan-sarbecovirus neutralization capacity retains their Omicron neutralization (5, 10) and could be used as cocktail therapy to contain the escape within conserved epitopes.

Here, to complement the lost interaction between SARS-CoV-2 VOCs Spike RBD (Delta and Omicron) and mAbs (CT-p59 and Etesevimab), we analyzed mutational space, van der Waal (vdW), and electrostatic surfaces complementarity, and structural stability parameters. Multiple derivatives of the antibodies were computationally generated and screened against Delta and Omicron variants to restore their binding affinities. The results in this study highlight the utility of our customized structure-based antibody design pipeline for the immediate selection of epitope-specific antibodies against emerging SARS-CoV-2 variants that could be validated *in vitro.* Additionally, the robustness of this protocol ensures broader application in response to future viral threats posed by rapidly emerging mutations.

## 2 Results

### 2.1 Single hotspot substitution in Spike can modulate the neutralization of nAbs

#### 2.1.1 CDRs composition and interface insights of CT-p59 and Etesevimab with RBD^WT^

**Figure 1:**
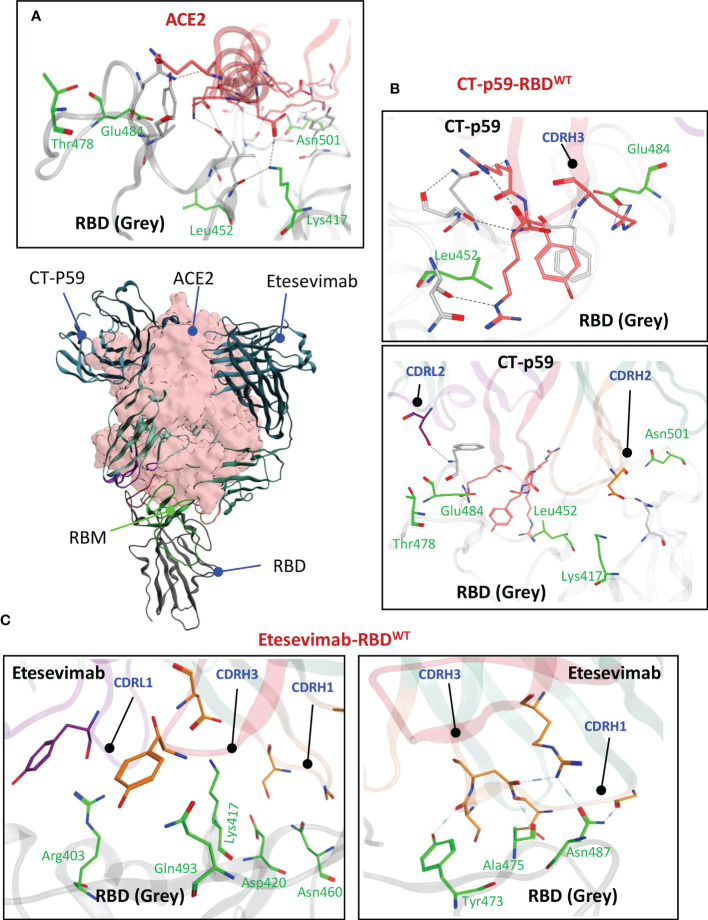
Monoclonal antibodies (mAbs) Etesevimab and CT-p59 neutralize SARS-CoV-2 and compete with ACE2 on RBD. **(A)** Both mAbs fully overlap with ACE2 and interact with receptor binding motif (RBM) in RBD. **(B)** CT-p59 utilizes its CDRs H2, H3 and L2 to engage with RBD. **(C)** Etesevimab binds RBD through its CDRs H1, H3, and L1. Amino acids in RBM that are shared by ACE, CT-p59 and Etesevimab are represented by green sticks and labeled green. Interface contacts along with their bonds-length, bonds-energies and bonds-type of both CT-p59 and Etesevimab are listed in **Tables S1** and **S2**.

The α1 helix in the peptidase domain of ACE2 is vital for viral binding and the optimal electrostatic potential and amino acid constitution in this helix are abused by the emerging SARS-CoV-2 variants to strengthen binding and enhance their transmissibility by mutating sub-optimal amino acids in the RBD. We have recently reported that Omicron (BA.1) enhances its ACE2 binding by substituting amino acids in the receptor binding motif (RBM) of RBD (5). The two mAbs selected for CDR diversification, *i.e.*, CT-p59 and Etesevimab, neutralize SARS-CoV-2 by fully overlapping with ACE2 at the RBM. Both mAbs share at least five amino acids Lys417, Leu452, Thr478, Glu484, and Asn501 with ACE2 in their epitopes on RBD (**Figure 1A**). CT-p59 utilizes CDRH2 and CDRH3 for RBD binding, whereas CDRH1 does not participate in the binding (**Figure 1B**). Beside hydrogen bonds by multiple residues, three salt bridges established by Arg403, Ly417, and Glu484 favor the CT-p59-RBD interactions (**Table S1**). Unlike CT-p59 where CDRH1 did not participate in antigen binding, Etesevimab binds RBD through CDRH1 and CDRH3. However, most of these contacts are hydrogen bonds, except Lys417 which established a salt bridges (bond energy=-29.03 kcal/mol) with CDRH3 Asp104 (**Figure 1C**; **Table S2**). Interface contacts along with their bonds-length, bonds-energies and bonds-type of both CT-p59 and Etesevimab are listed in **Tables S1** and **S2** and corresponds to **Figure 1**. Overall, these findings suggest that CT-p59 and Etesevimab have different compositions of CDRs and therefore respond differently in terms of their therapeutic efficacy against emerging SARS-CoV-2 variants.

#### 2.1.2 The escape mechanism of SARS-CoV-2 VOCs from CT-p59

**Figure 2:**
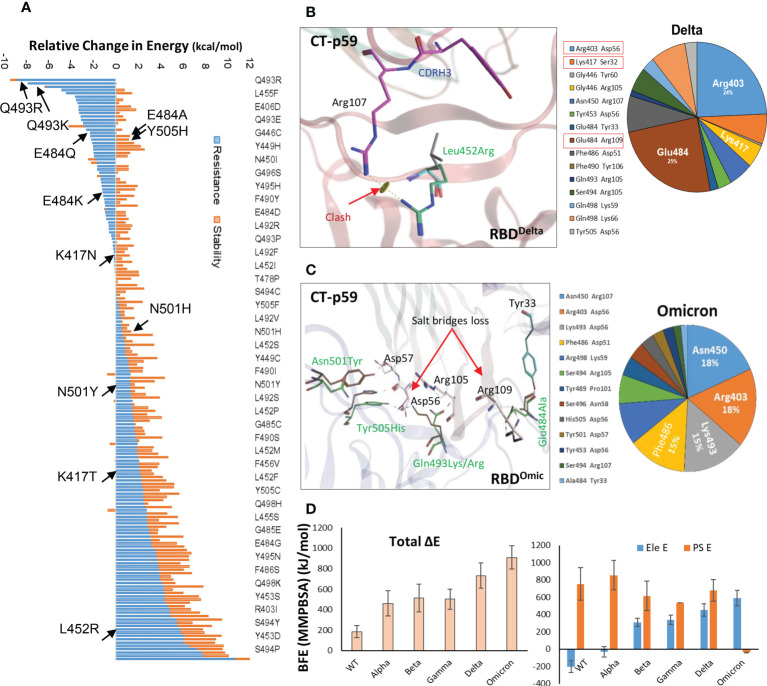
Immune escape and escape prediction of the SARS-CoV-2 VOCs. **(A)** SARS-CoV-2 immune escape prediction concerning CT-p59. The amino acid list on the right is shortened for clarity; affinity and stability changes for each of 176 residues corresponding to figure 2A are listed in **Table S3**. Amino acids and their respective substitutions involved in ACE2 and CT-p59 binding are labeled and indicated with black arrows. Blue bars indicate resistance to CT-p59, where negative values indicate increase in mAb-binding affinity and positive value indicate increase in mAb-escape or loss in affinity. Orange bars indicate stability in Ag-Ag complex. **(B)** Leu452Arg mutation in Delta VOC abolishes the electrostatic contacts between CDRH3 and RBD^Delta^. The pie chart represents the energy contribution (in percentage, unit is kcal/mol) at the RBD^Delta^-CT-p59 interface. **(C)** Glu484Ala mutation in RBD^Omicron^ abolishes the salt bridge between CDRH3 and RBD. The pie chart represents the energy contribution at the RBD^Omicron^-Etesevimab interface. Changes in the Interface contacts along with their bonds-length, bonds-energies and bonds-type of CT-p59 bound to WT, Delta ad Omicron variants, corresponding to figure 2A and C are listed in **Table S1**. **(D)** Free energy perturbation of the CT-p59-RBD complexes of all SARS-CoV-2 VOCs. Wild type and Alpha strains retain their electrostatic energy while other VOCs completely lost this potential.

Antibodies recognize distinct conformational epitopes on antigens that determine their specificity and binding affinity. Substituting a single hotspot residue in the epitope can abrogate mAb-antigen contact (4). To predict what future mutations in RBD would escape/increase CT-p59 neutralization (14), we constructed 176 single-mutation RBD muteins bound to CT-p59, and their relative binding energies were calculated to estimate mAb-resistance and mAb-RBD complex stability. Surprisingly, only 8 out of 176 substitutions, including Asn450Ile, Gly496Cys, Gln493Arg, Gln493Lys, Gln493Leu, Gly485Arg, Gly446Arg, and Gly446Ala in RBD were able to retain or enhance the binding strength of RBD^Mut^-CT-p59, whereas the rest of mutations weakened the stability of the mAb-RBD as well as increased resistance to CT-p59 (**Figure 2A**). Affinity change for each of 176 residues corresponding to **Figure 2A** are listed in **Table S3**. However, it is not possible to predict whether or not these mutations will occur following the same predicted order in the newly emerging strains of SARS-CoV-2. For example, Gln493Lys/Arg may enhance the CT-p59-RBD^Mut^ binding, while Leu452Arg in RBD is predicted to resist the CT-p59 neutralization. In line with these predictions, we could see that leu452Arg in the CT-p59-RBD^Delta^ model caused a clash with the CDRH3-Arg107 (**Figure 2B**), whereas Gln493Lys in the CT-p59-RBD^Omic^ established a strong salt bridge with CDRH2-Asp56, contributing 15% of the total binding energy at the interface (**Figure 2C**). However, Glu484Ala in CT-p59-RBD^Omic^ abolished the salt bridge with CDRH3-Arg109, which was contributing 22% and 25% of binding energy at the RBD^WT^-CT-p59 and RBD^Delta^-CT-p59 interfaces, respectively (**Figure 2B** and **Figure S1A**). Changes in the interface contacts along with their bonds-length, bonds-energies and bonds-type of CT-p59 bound to WT, Delta ad Omicron variants, corresponding to **Figures 2A, C** are listed in **Table S1**. These loss in electrostatic contacts, redistribution of the binding energies, and strong repulsion between the amino acids in CDRs and RBM are the possible causes that disrupt the neutralization of the Delta and Omicron variants. These results suggest that the fate of COVID-19 therapeutic mAbs relies on the serendipity of mutations in their epitopes on SARS-CoV-2 Spike, whereas mAbs that bind relatively conserved epitopes may retain their therapeutic ability, as we previously described (5). In addition, individual mutations in the RBD that retain or enhance the mAbs binding can be used in CDRs’ hotspots grafting for the designing of variant-escaped mAbs that share overlapping epitopes. To support the notion that the fate of COVID-19 therapeutic mAbs relies on the serendipity of mutations in their epitopes on SARS-CoV-2 Spike, whereas mAbs that bind relatively conserved epitopes may retain their therapeutic ability, further experimental and computational procedures could be performed.

#### 2.1.3 Free energies perturbations of the SARS-CoV-2 VOCs and CT-p59 complexes

To further support the above findings and expand the CT-p59 escape by other variants, we constructed 3D protein models of CT-p59 with all SARS-CoV-2 VOCs, including Alpha, Beta, Gamma, Delta, and Omicron and investigated their susceptibility and escape through free energy perturbations (FEP). Estimation of the End-point Molecular Mechanics/Generalized Born Surface Area (MMGBSA) energies revealed that CT-p59 may retain its neutralization against the Alpha variant while other variants had reduced the binding affinity, suggesting their potential neutralization escape (**Figure S1B**). We could validate this result by calculating MMGBSAs from the eight RBD^Mut^-CT-p59 and one RBD^WT^-CT-p59 complex with those of K_D_ values determined by Surface Plasmon Resonance (SPR) (**Figure S1C**). We further confirmed these energy terms by simulating the RBD^Mut^-CT-p59 complexes in a neutralized solvent condition and subjected to MMPBSA-based FEP. The binding free energies of all the VOCs increased by at least 2-fold (Alpha) and up to 5-fold (Omicron), losing their binding affinities (**Figure 2D**, *left*). The electrostatic potential energy (Ele E) was slightly retained (increased from -202 to -31 kcal/mol) by the Alpha variant but completely lost by other VOCs. Similarly, polar solvation energy (PSE) was retained by the Alpha variant, whereas the mutations in Beta, Gamma, and Delta strains dropped this energy term by 27-30% (**Figure 2D**, *right*). Due to multiple electrostatic residues substitution at the RBD^Omic^-CT-p59 interface, we could observe a peculiar shift between the Ele and PSE terms. The Ele E increased by 4-fold while the PSE shifted from positive (RBD^WT^-CT-p59 = 751 kcal/mol) to negative (RBD^Omic^-CT-p59=-39 kcal/mol), pointing towards the highly unstable energy state of the complex. These results are consistent with the recently reported experimental data demonstrating that CT-p59 retains its Alpha strain neutralization at reduced efficacy (14). However, in the case of Omicron this effect completely disappeared (15). Taken together, we suggest that at least one hotspot mutation within the epitope of CT-p59 is sufficient to drop, if not fully abolish, the neutralization capacity (*e.g.*, Delta), and that additional mutations can further help the escape (*e.g.* Omicron). However, this notion require further experimental support. The sharp decline in the efficacy of mAbs and nAbs due to emerging variants stresses the development of a robust and effective strategy to redirect these antibodies against emerging VOCs.

### 2.2 Affinity maturation of the VOCs-escaped mAbs

#### 2.2.1 CDR diversification and affinity maturation strategy

**Figure 3:**
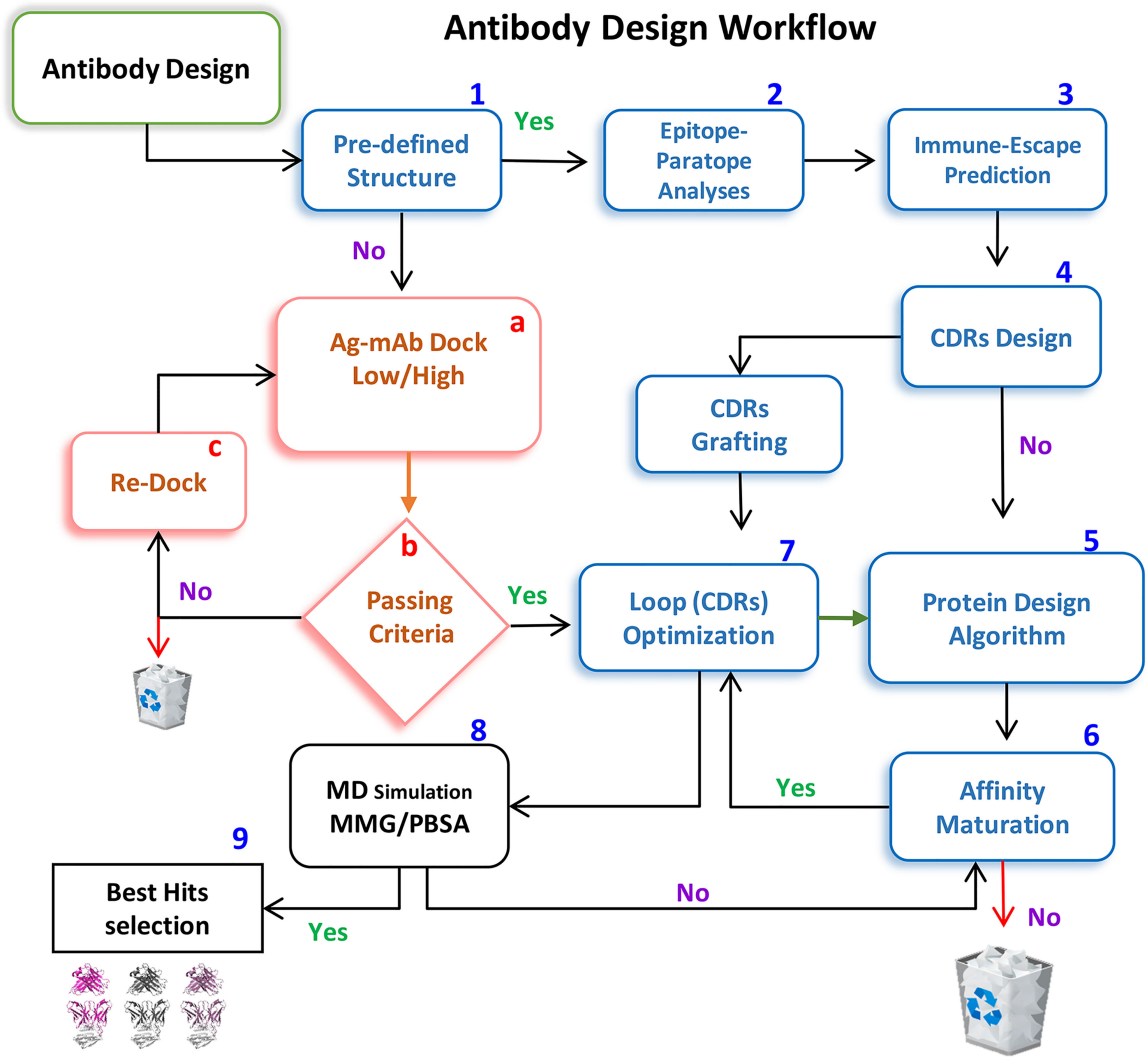
A workflow for repositioning SARS-CoV-2-escaped mAbs against emerging strains. Steps 1-9 are implemented to redesign CT-p59 against Delta and Etesevimab against the Omicron variant.

Single hotspot mutation within the epitope can substantially drops the mAbs’ neutralization whereas additional mutations can further help in escape (discussed above). Considering this scenario, CDR diversification can be exploited at its best to restore the binding affinity of the escaped mAbs by prudently substituting the lost hotspots within CDRs. We employed a computational method to re-engineer the escaped antibodies guided by the predefined hotspots-mediated epitope-paratope (Epi-Para) interactions. In particular, we considered common and crucial features of the dissociable protein partners in the Ab-Ag complex. After constructing the mAb-RBD^Mut^ complexes and optimization through MDS, we calculated the buried solvent-exposed surface and monitored interfaces including optimized vdW, electrostatic, and hydrogen-bond contacts at the Epi-Para junctions (discussed above). Second, the energy loss leading to the immune escape due to miss-paired hot spots was scrutinized by the previously described protein design methods (16). Third, we restored the lost contacts by reshaping the shape-complementarity of the disoriented CDRs by substituting the miss-paired hot spots at the Epi-Para interface. Hence, this strategy reshapes the dismantled Epi-Para interfaces by highly optimized original hot-spots-like interactions and optimally oriented CDRs with the best shape complementarity. The antibody-designing computational workflow is shown in **Figure 3**, where steps 1-9 are mostly implemented in this study.

#### 2.2.2 Delta-specific CT-p59 CDR diversification and affinity maturation

**Figure 4:**
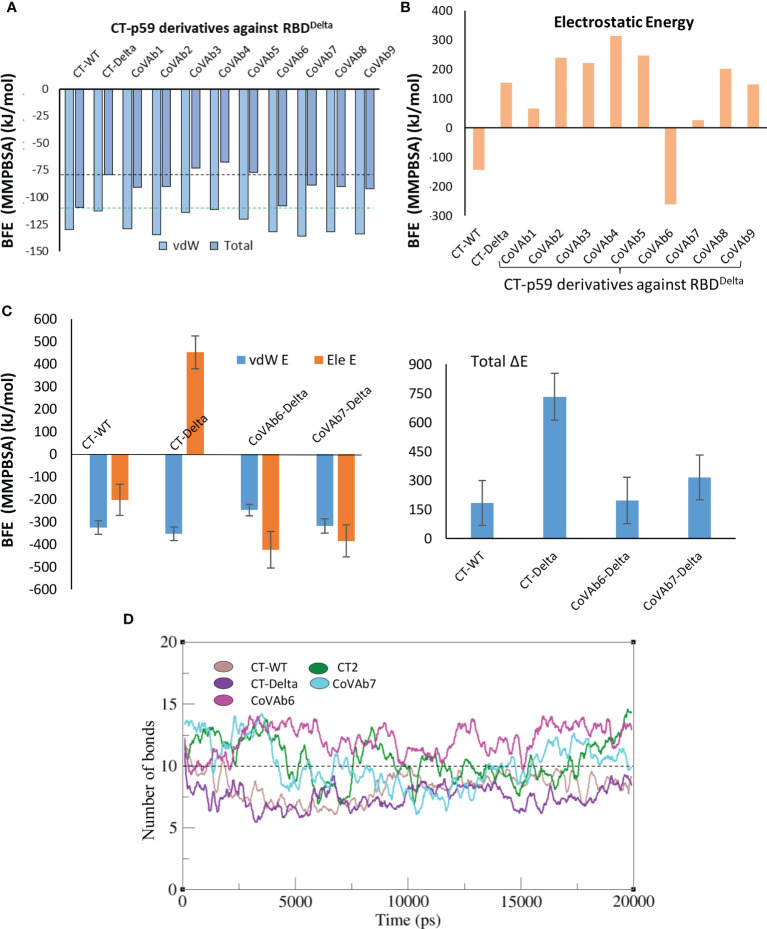
Binding free energy perturbation of the CDRs-diversified mAb (CT-p59) against Delta variant. **(A)** Relative changes in the total binding free energy of the CT-p59 derivatives (CoVAb1-9) are calculated through the MMGBSA method. **(B)** Relative change in the electrostatic energy (Ele) of the CT-p59 derivatives (CoVAb1-9). CoVAb6 fully and CoVAb7 partially restore their Ele potential that was lost by CT-p59 against the Delta variant (CT-Delta). **(C)** Binding free energy perturbation (MMPBSA method) of the top hits from CDR-diversified mAbs. **(D)** Change in the number of hydrogen bonds at the RBD-mAbs interface. The lowest number of bonds was recorded in CT-Delta. (CT-Delta=CT-p59-RBD^Delta^). CoVAbs are CDR-diversified CT-p59 derived mAbs against the Delta variant.

We extracted multiple low-energy conformers of the CT-p59-RBD^Delta^ complex from the MDS trajectory and investigated the interface changes as discussed above (**Figure 2A**). Considering all CDRs but CDRL1 for diversification, we monitored the relative changes in energies for each mutations and their effects on change in binding affinities of CT-p59-derivatives and RBD^Delta^ (**Table S4**). Single substitutions in the CDRs that restored or compensated the lost Epi-Para contacts and enhanced the binding affinities were combined in CT-p59-derivatives and their combined effect on binding was investigated. All three CDRs in the heavy chain variable domain had a significant impact on CT-p59-RBD^Delta^ complex stability. We selected nine CT-p59 derivatives (e.g. CoVAb1-9) and calculated their end-point binding free energies through MMGBSA against RBD^Delta^. Mutations carried in the CDRs of CoVAb1-9 are listed in **Table S5**. CoVAb3, 4, and 5 had reduced total binding energies while the other six derivatives had better binding strength compared to that of CT-p59-RBD^Delta^ (**Figure 4A**). Only CoVAb6 restored its total and vdW energies (-131 kcal/mol) similar to that of CT-p59-RBD^WT^ (-129 kcal/mol). However, the electrostatic potentials of all CoVAbs, except CoVAb6 and CoVAb7, remained similar to that CT-p59-RBD^Delta^. CoVAb6 had a dramatic shift in its Ele potential (dropped from 154 kcal/mol to -260 kcal/mol) and acquired overall energy terms similar to that of CT-p59-RBD^WT^ (**Figure 4B**).

To demonstrate whether mutations in CT-p59 CDRs enhance the binding affinity against RBD^WT^, we generated mutations in all three heavy chain CDRs and CDRL2 and calculated their per residues change in binding energies against RBD^WT^ (**Table S6**). CT-p59 derivatives against RBD^WT^ were generated by combining high-affinity mutations for CT-p59 derivatives. According to the MMGBSA-based end-point energy calculation, CT2 derivative showed slightly better total binding energy (-126.08 kcal/mol) whereas the rest of the derivatives CT1, CT3, and CT4 showed similar or lower binding energies as compared to parent CT-p59 (-109.45 kcal/mol) (**Figure S2A**). This suggests that residues within the CDRs that have already matured for the cognate epitopes may lose their binding potential if substituted.

As MMGBSA calculates the binding energy on a single frame (in this case) and MMPBSA takes the entire trajectory into account containing hundreds of structural frames that evolve during time, we used MMPBSA to validate the binding affinities of CoVAb6 and CoVAb7 against RBD^Delta^. Due to loss in electrostatic contacts and the establishment of the steric clash between Arg107 of CT-p59 and Arg452 of RBD^Delta^ the Ele E had a vivid shift from –ve (RBD^WT^=-202 kJ/mol) to +ve (RBD^Omic^=451 kJ/mol) (**Figure 4C**, *left*). Although the MMGBSA-based energy calculation showed that this loss in Ele potential was fully restored in CoVAb6 and partly in CoVAb7, MMPBSA energy perturbation confirmed that both CoVAb6 and CoVAb7 had substantially restored Ele E potential. The total binding energy was dropped from 732 kJ/mol in CT-p59-RBD^Delta^ to 196 kJ/mol in CoVAb6 and 316 kJ/mol in CoVAb7, where that of CT-p59-RBD^WT^ was 184 kJ/mol (**Figure 4C**, *right*). Interestingly, we found that CoVAb6-RBD^Delta^ had similar total energies as that of CT-p59-RBD^WT^ in both MMGBSA- and MMPBSA-based calculations. The total number of hydrogen bonds was the lowest in CT-p59-RBD^Delta^, which were restored or increased in both CoVAb6-RBD^Delta^ and CoVAb7-RBD^Delta^ as compared to CT-p59-RBD^WT^ (**Figure 4D**). Together, these data suggest that CDR diversification can restore the lost electrostatic potential and further enhance the binding strength of modified mAbs against the escaped antigens.

#### 2.2.3 CoVAb6 and CoVAb7-RBD^Delta^ interface assessment

**Figure 5:**
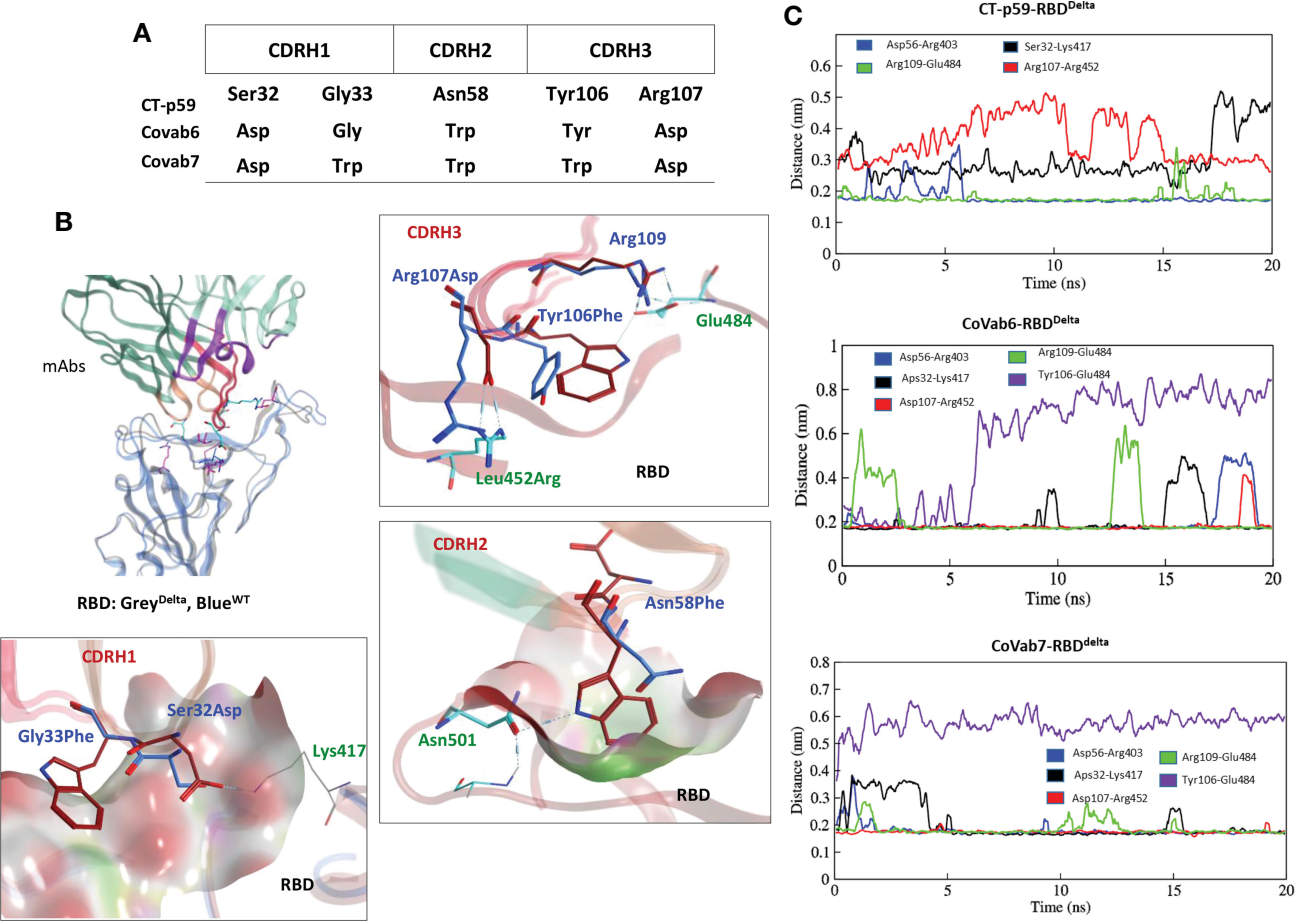
CDR diversification and interface analysis of the CoVAb-RBD complexes. **(A)** Contact favorable mutations created in CT-p59 CDRs to restore their efficacy against the Delta variant. **(B)** Interface analysis of the CoVAb6-RBD and CoVAb7-RBD complexes. Salt bridges between Arg452 and Asp107 are restored after Arg107Asp substitution in CDRH3 of CT-p59. Interface contacts along with their bonds-length, bonds-energies and bonds-type of CoVAb6-RBD^Delta^ and CoVAb7-RBD^Delta^, corresponding to figure 5B are listed in **Table S7**. **(C)** Changes in the hydrogen bonds and salt bridges during simulation.

Five potential hotspots substitution restore the affinity of CT-p59 against escaped the Delta variant (**Figure 5A**). Among them, Arg107Asp in the CDRH3 was the most crucial substitution, changing the steric clash between Arg107 and Arg452 in RBD^Delta^ into a stable salt bridge and restored the lost electrostatic potential in CoVAb6 and CoVAb7 (**Figure 5B**). Interface contacts along with their bonds-length, bonds-energies and bonds-type of CoVAb6-RBD^Delta^ and CoVAb7-RBD^Delta^, corresponding to **Figure 5B** are listed in **Table S7**. To validate the stability of salt bridge between Asp107 and Arg452 of RBD, the distances of Arg452 concerning CT-p59^Arg107^, CoVAb6^Asp107^, and CoVAb7^Asp107^ were tracked as a function of time. We observed a clear shift from unstable (CT-p59^Arg107^) to stable salt bridge (CoVAb6^Asp107^, and CoVAb7^Asp107^) over 20 ns trajectory (**Figure 5C**). In addition, the distance between CT-p59^Ser32^ and Lys417 of RBD^Delta^ was 3 Å with some rigor, which was lost during the last quarter of simulation. Ser32Asp substitution in the CDRH1 of CoVAb6 and CoVAb7 established a stable salt bridge with Lys417 in RBD^Delta^, which remained intact at ~2 Å. We found that aliphatic and aromatic amino acids were the best fits to enhance the complementarity and CDRs fitting onto the epitope (**Figure 5B**). Single Asn58Trp substitution in the CDRH2 of CoVAb6 was sufficient to restore its binding; however, triple Trp substitution (Gly33Trp, Asn58Trp, and Tyr106Trp) in all three CDRs enhanced the vdW potentials in CoVAb7 (**Figure 4C**). However, this additional Trp supplementation increased the total binding energy from 196 kJ/mol in CoVAb6 (Asn58Trp substitution only) to 315 kJ/mol in CoVAb7 (Gly33Trp, Asn58Trp, and Tyr106Trp substitutions), suggesting that overabundance of bulky hydrophobic amino acids may compromise the CDR-fitting onto epitopes. Taken together, electrostatic and aliphatic amino acids substitutions in CoVAb6 and CoVAb7 restored their binding strength against RBD^Delta^ and possibly their neutralization as well, which may require further validation through pseudovirus neutralization or biophysical assays such as SPR.

#### 2.2.4 Omicron-specific Etesevimab CDRs diversification and affinity maturation

**Figure 6:**
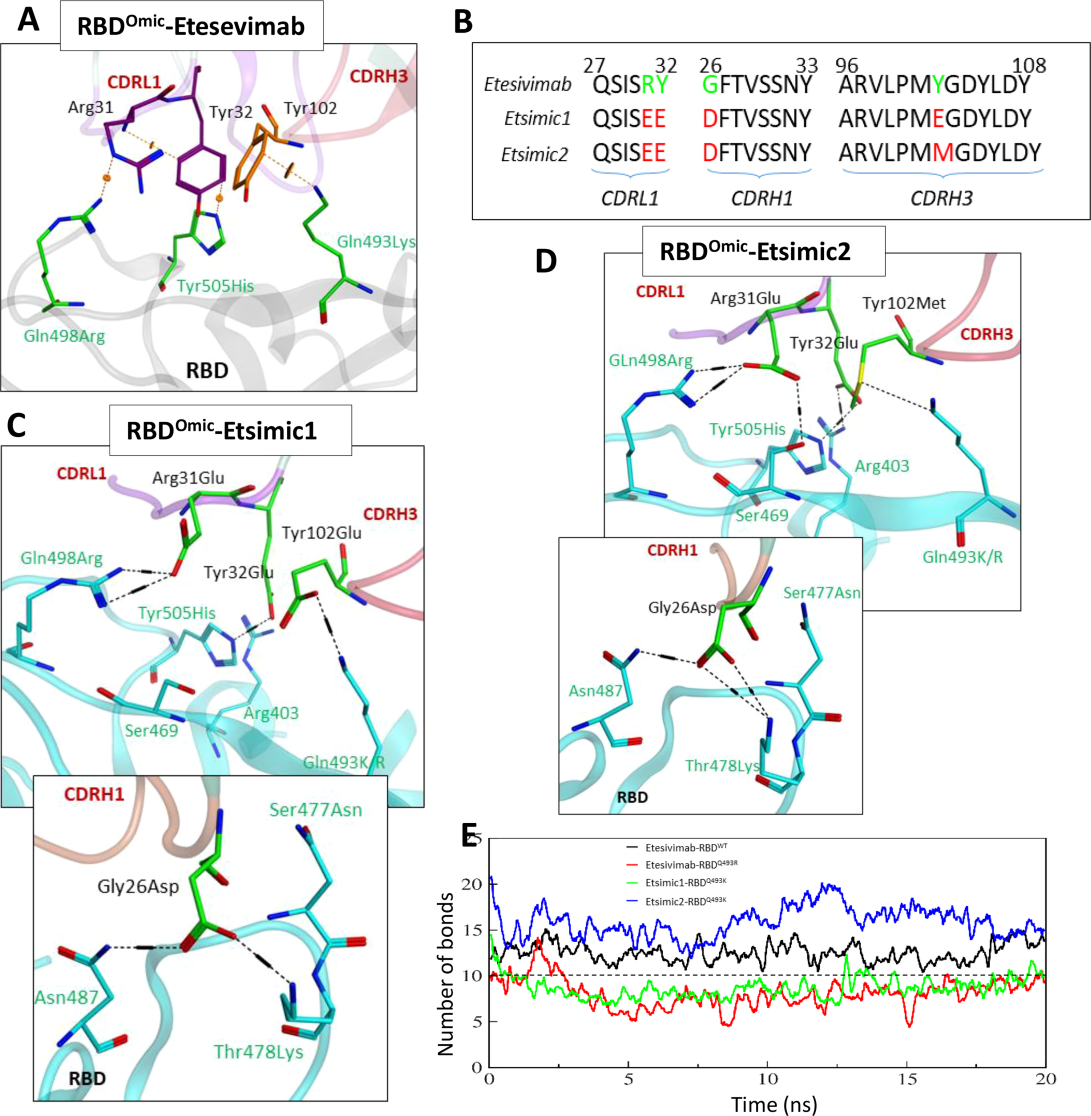
Interface analyses and CDR diversification of the Etesevimab against the Omicron variant. **(A)** Three mutations in the RBD^Omic^ i.e. Gln493Lys, Gln498Arg, and Tyr505His, abolish vdW contacts at the Etesevimab-RBD^Omic^ interface. **(B)** Contact favorable mutations created in Etesevimab CDRs (L1, H1, and H3) to restore their efficacy against the Omicron variant. **(C, D)** Interface analyses of the Etesevimab derivatives (Etsimic1 and Etsimic2) and RBD complexes. Hydrogen bonds are restored and salt bridges are established after CDRL1-Arg31Glu, CDRL1-Tyr32Glu, CDRH1-Gly26Asp, and CDRH3-Tyr102Glu/Met substitutions. Interface contacts along with their bonds-length, bonds-energies and bonds-type of Etsimic1-RBD^Omic^ and Etsimic2-RBD^Omic^, corresponding to figure 6B and 6D are listed in **Table S8**. **(E)** Change in the number of hydrogen bonds at the RBD-mAbs interface. The lowest number of bonds was recorded in Etesevimab-RBD^Omic(BA.1)^.

As discussed above, Etesevimab largely utilizes its CDRH1 and CDRH3, and partly CDRL1 to bind RBD (see **Figure 1C**). We sought to deduce the effect of RBD mutations reported in the Omicron variant on the Etesevimab escape by constructing a 3D Etesevimab-RBD^Omic^ structural model. Unlike CT-p59-RBD^Omic^ which has no significant steric clashes due to miss-paired Epi-Para contacts, CDRL1 and CHRH3 in Etesevimab clashed with all the three mutated amino acids (i.e. Gln498Arg, Tyr505His, and Gln493Lys) in RBD^Omic^. Of note, Gln498Arg created a steric clash as well as a repulsive electrostatic environment around Arg31 in CDRL1, which was aided by the π-cation clash between Gln493Lys of RBD^Omic^ and Tyr102 in CDRH3 (**Figure 6A**).

As with CoVAbs, we designed several Etesevimab derivatives and selected Etsimic1 and Etsimic2 as the best candidates to restore RBD^Omic^ binding and possibly SARS-CoV-2 neutralization. Four amino acids substitutions in CDRL1, CDRH1, and CDRH3 not only restored the binding affinity in Etsimic1 and Etsimic2 but also surpassed that of Etesevimab-RBD^WT^ (E total=173 kJ/mol). Both Etsimics differ at single residue where Tyr102 in Etesevimab was substituted by Glu102 in Etsimic1 and Met102 in Etsimic2 (**Figure 6B**). Asp26 in CDRH1 of Etsimic1 was optimally oriented and established a salt bridge with Thr478Lys and a hydrogen bond with surrounding Asn87 in RBD^Omic^ (**Figure 6C**). The EEE substitutions (i.e. Arg31Glu, Tyr32Glu) in CDRL1 and Tyr102Glu mutations in CDRH3 created a network of salt bridges around the Glu498Arg, Tyr505His, Glu493Lys, and Arg403 in RBD^Omic^. We could observe a similar network in Etsimic2-RBD^Omic^ (**Figure 6D**). Interface contacts along with their bonds-length, bonds-energies and bonds-type of Etsimic1-RBD^Omic^ and Etsimic2-RBD^Omic^, corresponding to **Figures 6B, D** are listed in **Table S8**.

Next, we monitored the stability of these bonds as a function of time, revealing a rigorous fluctuation between Arg31-Gln498Arg, Tyr102-Tyr505His, and Gly26-Lys478 pairs caused by the steric clashes in Etesevimab-RBD^Omic^ complex (**Figure S3A**). Upon residue substitution, we found a substantial reduction in the minimum bond lengths (dropped from ~8Å in Etesevimab to ~4Å in Etsimics). The clash between Arg31Glu-Arg498 pairs disappeared, establishing a steady salt bridge (bond length ~2Å) in both Etsimics with RBD^Omic^ (**Figures S3A, B**). In addition, Gly26Asp in CDRL1 established a steady salt bridge with Lys478, which was otherwise absent in Etesevimab-RBD^WT^. The total number of hydrogen bonds elevated to ~15 in Etsmic2-RBD^Omic^ from ~12 in Etesevimab-RBD^WT^, which was dropped to ~6 in Etesevimab-RBD^Omic^ (**Figure 6E**).

**Figure 7:**
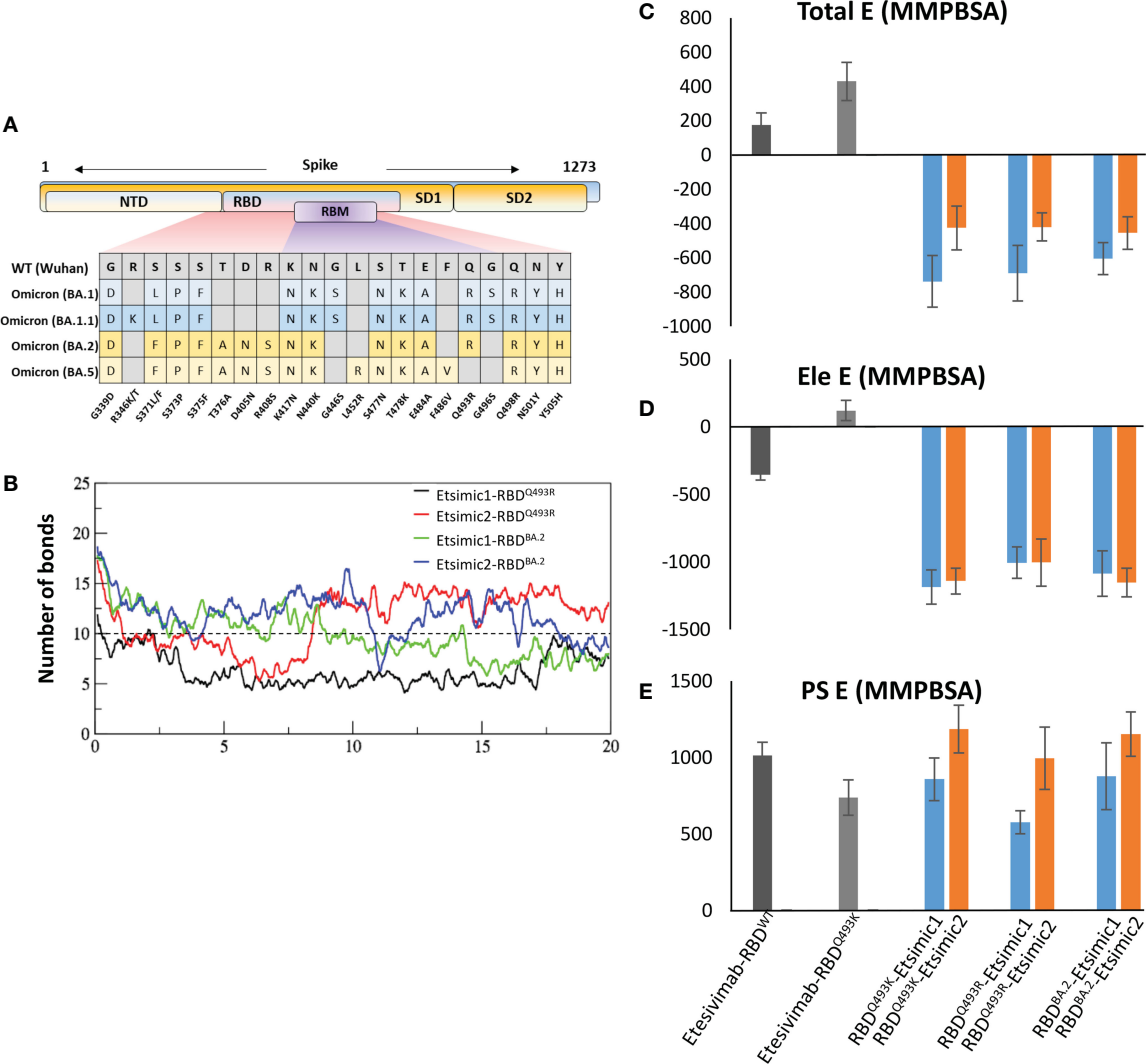
Binding free energy perturbation of the CDRs-diversified mAb (Etsimics) against Omicron BA.1 and BA.2 variants. **(A)** Mutations within the RBD domain of Omicron sub-variants concerning the Wuhan strain. **(B)** Change in the number of hydrogen bonds at the RBD^BA.2^-Etsimic interface. **(C-E)** Relative change in the total binding free energy of the Etesevimab derivatives as calculated through the MMGBSA method.

The BA.1 lineage of the Omicron variant was found to have either Gln493Lys (non-dominant) or Gln493Arg (dominant variant) (17) and, during this study new sublineages of Omicron BA.2, BA.3, and BA.4/5 appeared, where BA.2 became the dominant variants very quickly (18). The differences and similarities in the RBD of these lineages are depicted in **Figure 7A**. We sought whether Gln493Lys to Gln493Arg mutation in BA.1 and differences in the BA.1 and BA.2 affect the binding affinity of Esimic1 and Esimic2. The total number of hydrogen bonds was first investigated as a function of time, which slightly dropped in Etsimic1-RBD^Q493R^; however, Etsimic2-RBD^Q493R^, Etsimic1-RBD^BA.2^, and Etsimic2-RBD^BA.2^ retained this number (**Figure 7B**). Interestingly, relative binding FEP analysis revealed that both Etsimic1 and Etsimic2 retained their binding strength against BA.2 and BA.1 (RBD^Q493R^) variants, where Etsimic1 had relatively better affinity compared to Etsimic2 (**Figures 7C-E**). This suggests that Etsimics can withstand slight variations in their epitopes and have the potential to neutralize the sublineages of Omicron, although requiring further experimental validation.

## 3 Discussion

The abrogation of Spike-ACE2 is considered one of the promising COVID-19 therapeutic strategies to block the virus at an early stage of infection. Various nAbs have been approved by FDA for this purpose and many small molecules and peptides are under clinical and pre-clinical evaluation (19–22). Studies have been conducted to probe the antigenicity of the SARS-CoV-2 Spike protein by mapping several overlapping epitopes using structural (23–25) and deep mutational scanning techniques (26). Strikingly, over 90% of the convalescent plasma and serum-derived SARS-CoV-2 nAbs target the Spike RBD (27), and most of the FDA-approved RBD-binding nAbs are evaded by the emerging VOCs (5).

Antibodies screening and selecting high-affinity molecules during this process is a hectic task that requires expensive and often time-consuming procedures like affinity maturation, epitope mapping, and binding kinetics of the Ab-Ag complexes (6). The swift failure of the drugs due to viral mutations has put the fate of COVID-19 therapeutics, particularly nAbs, and vaccines in jeopardy. As discussed above, a single mutation in the antigenic epitope can render effective antibodies incapable of neutralization. With the advent of structural and computational biology, the knowledge of pre-defined hotspots-mediated epitope-paratope interfaces could be utilized to redirect/design escaped antibodies against the emerging SARS-CoV-2 variants. Substituting the concerned hotspots in CDRs into structurally favorable and contact-restoring amino acids can help in redirecting the escaped antibodies against new variants. High-resolution structural models of both antigen and antibodies are therefore vital in this regard, as lack of exact interface knowledge may hinder the CDRs diversification rationale.

Computational approaches such as OptMAVEn (28) and AbDesign, utilizing a dock-and-design strategy, generate ensembles of docked conformers followed by tightly bound top-ranked Ab-Ag poses and allow for CDR optimization (see **Figure 3**). However, the success of precise antigen positioning is constrained by the accuracy of scoring methods and sampling algorithms of the computational tools, which often prioritize the desired binding patches on a static antigen to the antibody scaffold. This biased antigen positioning could be overcome by an induced-fit docking algorithm that allows, to some extent, the mobility of epitope residues during docking. Conformational changes at the Epi-Para interface of the rigid-body docking procedure may lead to a reorientation of Ab-Ag poses, abominating complexes such as the Prb-Pdar protein complex (29). Therefore, it is important to consider the pre-defined interaction patterns of the cognate binders for CDRs restoration to ensure the favorability of desired binding mode and stability in the solution state.

In this study, we computationally redesign the mAbs targeting disoriented pre-defined epitopes for the immune-escaped Delta and Omicron variants. We established a new approach that entails the heuristic design strategy to some extent and emulates the biased pose-prioritization binding of the docking algorithms. This method involves the reshaping of miss-paired Epi-Para hotspots by substituting amino acids in the corresponding CDRs to accommodate mutations in RBD and regain the lost binding affinity. In addition, in-solvent simulations confirm the stability and resistance of the Ab-Ag poses to conformational changes and reorientation.

Similar techniques, overlapping with the one propose in our study, have been recently put into practice by AstraZeneca, one of the lead COVID-19 fighting companies (30). Here they used a mere computational model of the hybridoma-derived antibody AB1 and its antigen muCCL20 to fine-tune their Epi-Para interface through Ab-Ag docking and in silico alanine mutagenesis, which allowed them identifying two single-point mutations that increased the physical binding affinity (estimated by SPR) of the AB1 derivatives C1 and C1-16 against muCCL20 by four fold (KD=2.3 nM and KD=2.8 nM, respectively) as compared to AB1-muCCL20 affinity (KD=9.1 nM). Reinforcing the in silico antibody designing strategy, a SARS-CoV-1 neutralizing nAbs were redirected against SARS-CoV-2 very recently, by computationally engineering their CDRs and confirming their neutralization *in vitro* and binding affinity through bilayer interferometry (31). Here the crystal structures of SARS-CoV-bound mAbs were investigated for conserved epitopes compared to SARS-CoV-2, and their CDRs were redesigned through computational affinity maturation using the criteria of shape-complementarity, buried solvent-accessible surface area, and number of unsatisfied polar atoms, which is considerably overlapping with the acquired in our study. Out of several hits, D27 (mAb) was found to bind SARS-CoV-2 RBD at KD=177 nM whereas none of its parent mAbs including S230, 80R, m396, and F26G19 showed any cross reactivity with SARS-CoV-2 RBD (31).

Conclusively, the examples of CoVAbs and Etsimics identified here put forth the idea of utilizing computational tools in mAbs design as a viable strategy to regain the specificities and binding affinities of existing antibody scaffolds, although further experimental validation is required. The structure-based design of antibodies with improved computational accuracy and parallel optimization *via* MDS and FEP provides a complementary and time-efficient method for the fast development of SARS-CoV-2 therapeutic antibodies to meet the demand to combat emerging immune-escaping VOCs.

## 4 Methods

### 4.1 Protein structures modeling

The structural coordinates of the proteins used in this study were obtained from the PBD databank, including the following: ACE2- RBD^WT^ (PDB ID: 6MOJ), ACE2-RBD^Omic (BA.1)^ (PDB ID: 7WBP), CT-p59-RBD^WT^ (PDB ID: 7CM4), Etesevimab-RBD^WT^ (PDB ID: 7C01), RBD^Alpha^ (PDB ID: 7R15), RBD^Beta^ (PDB ID: 7NXA), RBD^Delta^ (PDB ID: 7WBQ). The structural coordinates constructed by the replacement process are as follows. RBD^Gamma^ was constructed by mutating respective amino acids in RBD^Beta^ (PDB ID: 7NXA). RBD^Omic (BA.2)^ was constructed by mutating respective amino acids in RBD^Omic (BA.1)^ (PDB ID: 7WBP). The heterotrimeric structure of RBD^WT^ with CT-p59 and Etesevimab was constructed by superimposing RBD in Etesevimab-RBD^WT^ and CT-p59-RBD^WT^ complexes. CT-p59-RBD^Delta^ was built by replacing RBD^WT^ in CT-p59-RBD^WT^ with RBD^Delta^. Similarly, CT-p59-RBD^Omic^ was built by replacing RBD^WT^ in CT-p59-RBD^WT^ with RBD^Omic^. Etesevimab-RBD^Omic^ was built by replacing RBD^WT^ in Etesevimab-RBD^WT^ with RBD^Omic (BA.1)^ and RBD^Omic (BA.2)^. Free BIOVIA Discovery Studio Visualizer was used for constructing mutant RBD, (http://www.accelrys.com). The structural coordinates of manually built models were relaxed by solvating in a cubic box filled with TIP3P water model and energy minimizing in GROMACS 2020 (32) under CHARMM37 force field (33) following a steep descent algorithm.

### 4.2 Escape prediction and CDR diversification

Antibodies in CT-p59-RBD^WT^ and Etesevimab-RBD^WT^ complexes were annotated as described previously (34, 35). The resistance of RBD^Mut^ towards CT-p59 or Etesevimab was determined through a resistance scan package in MOE 2022 (Chemical Computing Group, Montreal CANADA). One limitation of this approach is that the predicted resistance is limited to the antibody under study and predicted mutation in the RBD will confirm the immune escape of a new variant concerning the same antibody. As the composition of CDRs differs in antibodies, neutralizing antibodies binding to the non-overlapping epitopes on RBD may respond differently to the escape prediction model here. We mutated all residues within 10Å of the CDRs using Unary Quadratic Optimization (UQO) model under the molecular dynamics ensemble that estimates the stability of the output conformers after 1 ps run of simulation at constant temperature (36). The output conformers were saved in a database and ranked according to the resistance and instability criteria. Mutants were considered resistant if the relative change in binding energy was equal to or more than 1.0 kcal/mol. Similarly, all mutants were placed as unstable when the relative change in Ab-RBD^Mut^ was equal to or more than 1.0 kcal/mol. Mutants with more unstable energy but lower resistance were also placed in escape mutants.

For CDRs diversifications we used overlapping criteria; nonetheless, to get better insights about the disoriented Epi-Para interface and miss-paired hotspots in RBD^Mut^-mAbs, we created electrostatic and vdW (interaction) surfaces at the Epi-Para interfaces using APBS and APBSrun plugins in VMD. Suboptimal residues and those with prominent steric clashes were identified. The hotspots and suboptimal amino acids and their solvent surface exposure were examined through PDBePISA (37) and validated through DrugscorePPI (38). Considering the surface complementarity, miss-paired amino acids, hotspots, and suboptimal residues, CDRs were diversified using a protein design package in MOE 2022. Initially, the CDRs in RBD^Mut^-mAbs were subjected to single residues substitution and the mutants were sorted and ranked based on high binding energies, as described previously (39). Second, the top-ranked single CDR-mutants were selected and combined in multiple-CDR mutants, producing mAbs with enhanced RBD^Mut^ binding and Epi-Para complementarity. An overall protocol is outlined in **Figure 3**.

### 4.3 Molecular dynamics simulations

All protein models were simulated in a separated cubic box saturated with the TIP3P solvent model. The boundaries of the box were 10Å apart from the centralized protein models that were neutralized with Na^+^ and Cl^-^ ions and extra 0.1M NaCl concentrations. All systems were first energy minimized as discussed above and then equilibrated under constant temperature (NVT) and constant pressure (NPT) conditions for 0.5 ns. To keep the systems from breaking, proteins and solvents were separated and constraints were applied to protein atoms. During the NVT step, the temperature was coupled with the v-rescale (modified Berendsen thermostat) method while the unmodified Berendsen algorithm was used in the NPT step (40). All systems were simulated for at least 20 ns with no structural constraints. The long-range electrostatic interactions were computed by utilizing the Particle Mesh Ewald algorithm (41). After completion of the simulation, the artifacts were removed from the MD trajectories using –PBC and –fit flags implemented in the trjconv tool with different functions including whole, nojump, and rot+trans. For trajectories analyses, built-in options in GROMACS including rmsf, rms, mindist, and hbond were used.

### 4.4 Free energy perturbation and binding free energies calculation

For binding energy calculations, we used endpoint binding free energy MMGBSA method using HawkDock server (42) and free energy perturbation methods using MMPBSA implemented in GROMACS (v 5.0 and earlier) (43), which is best suited for energy calculation of the different ligands bound to the same target. The newer version of GROMCS is not compatible with MMPBSA, thus, the topology files for each Ab-Ag complex were generated through v 5.0. The optimized simulation trajectory containing 100 frames was analyzed for binding free energies calculation as described previously (44).

## Data availability statement

The original contributions presented in the study are included in the article/**Supplementary Material**. Further inquiries can be directed to the corresponding authors.

## Author contributions

MS and HW contributed to the conceptualization of the project. MS designed the methodology. MS, J-YS, and HW wrote the original manuscript draft. HW supervised the study and provided funding acquisition. All authors contributed to the article and approved the submitted version.
