# Supplementary material for: Rational strategies for enhancing mAb binding to SARS-CoV-2 variants through CDR diversification and antibody-escape prediction
Source: Front Immunol. 2023 Mar 31;14:1113175. doi: 10.3389/fimmu.2023.1113175 (PMC10102385; doi:10.3389/fimmu.2023.1113175)
Supplement: Supplementary file 1 [file DataSheet_1.pdf]

Development of therapeutic antibodies against escaped SARS-CoV-2 VOCs by the CDRs-diversification of FDA-approved mAbs

Masaud Shah[1], Ji-Yon Shin[1, 2], and Hyun Goo Woo[1, 3,*]

[1]Department of Physiology, Ajou University School of Medicine, Suwon 16499, Republic of Korea

[2]Korea Initiative for fostering University of Research and Innovation (KIURI) Program, Ajou University School of Medicine, Suwon 16499, Korea

[3]Department of Biomedical Science, Graduate School, Ajou University, Suwon 16499, Korea

*__Corresponding author__

Hyun Goo Woo, M.D., Ph.D.

Tel: 82-31-219-5045,

Fax number: 82-31-219-5049,

E-mail address: hg@ajou.ac.kr

Supplementary figures

Supplementary Figrues

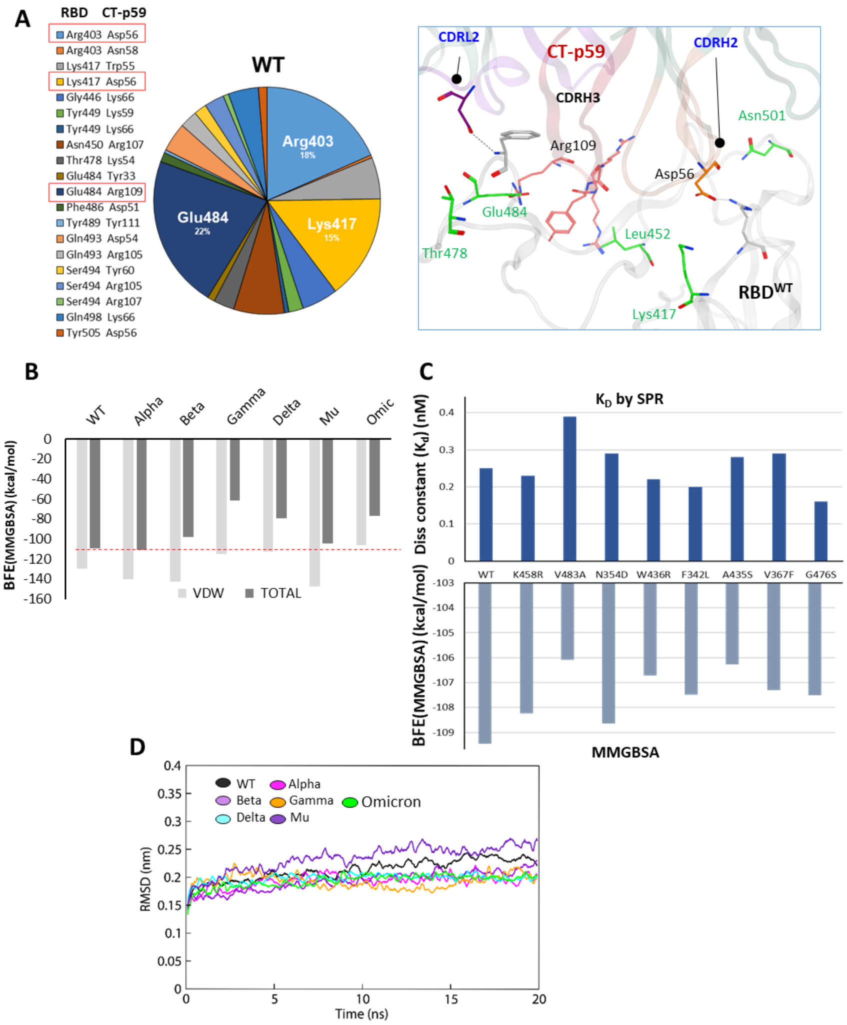

Figure S1: Interface and binding affinity of the CT-p59 with SARS-Cov-2 VOCs. A) Amino acids pairs involved at the interface of RBD^{WT}-CT-p59 and their respective energy contribution (pie-chart, kcal/mol) in percentage. Residues with the highest binding energies are labeled and displayed in the cartoon representation. **B**) Change in the binding energy (MMGBSA) as CT-p59 binds VOCs. **C**) Validation of the change in binding energy (MMGBSA). Binding affinity results taken from SPR and HawkDock servers are compared. **D**) Change in the root mean square deviation of the CT-p59-RBD (all VOCs) complexes.

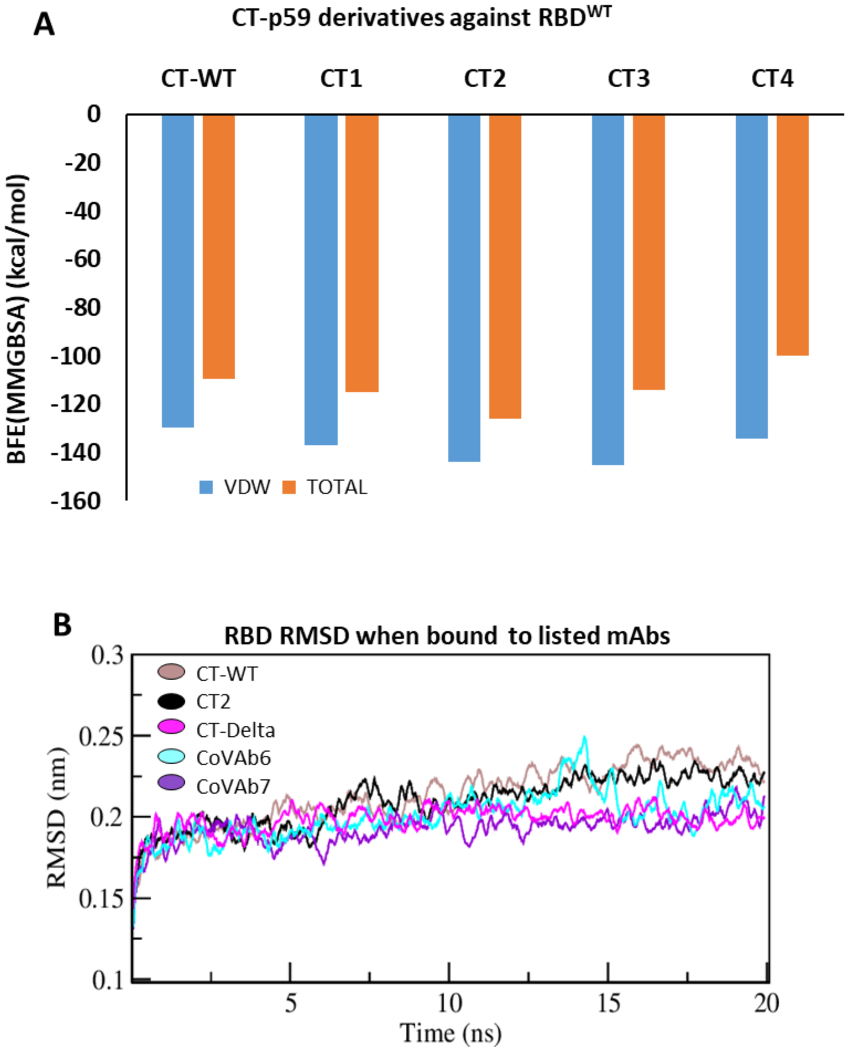

Figure S2: Binding affinity of the CDRs diversified mAbs against WT and Delta strains. A) Relative change in the binding energy (vdW and total energies in kcal/mol) of CDRs-diversified mAbs (CT1-4) against WT stain. CT-WT is CT-p59-RBD^{WT}. **B**) Change in the root mean square deviation of the CDRs-diversified CT-p59 derivatives against RBD^{WT} and RBD^{Delta}. CT-Delta is CT-p59-RBD^{Delta}. CoVAbs are CDR-diversified mAbs against RBD^{Delta}.

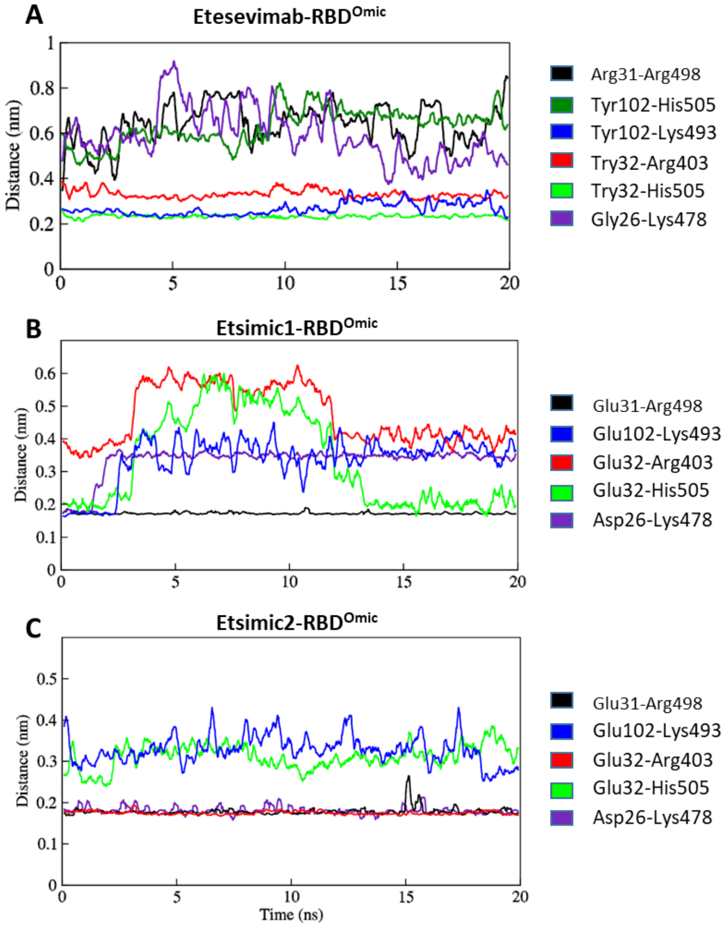

Figure S3: Change in the minimum distances and salt bridges between epitope-paratope amino acids pairs of Etesevimab and its CDRs-diversified derivatives Etsimic1 and Etsimic2 with respect to $RBD^{Omicron}$.

Supplementary Tables

Table S1. Comparative interface of CT-P59 with RBD of wild type, Delta, and Omicron (BA.1) Variant of SARS-CoV-2.

CT-p59-RBD(WT)						CT-p59-RBD(Delta)						CT-p59-RBD(Omicron)					
**Type**	**CT-p59**	**RBD(WT)**	**Energy**	**Dist**	**BB**	**Type**	**CT-p59**	**RBD(Delta)**	**Energy**	**Dist**	**BB**	**Type**	**CT-p59**	**RBD(Omicron**	**Energy**	**Dist**	**BB**
H	Tyr33	Glu484	-1.6	2.7	b	H	Ser32	Lys417	-7.2	2.84	--	H	Tyr33	Ala484	-1	2.92	b
H	Asp51	Phe486	-2.1	2.8	b	H	Tyr33	Glu484	-2.1	2.75	b	H	Asp51	Phe486	-8.4	2.98	b
H	Asp54	Gln493	-6.4	2.91	--	H	Asp51	Phe486	-8.7	2.79	b	I	Asp56	Arg403	-10.72	3.32	--
H	Lys54	Thr478	-4.6	2.88	--	**IH**	**Asp56**	**Arg403**	**-26.88**	**3.06**	**--**	H	Asp56	Tyr453	-1.5	2.85	--
H	Trp55	Lys417	-9.2	3	b-	H	Asp56	Tyr453	-3.5	2.7	--	**IH**	**Asp56**	**Lys493**	**-9.07**	**3.15**	--
**IH**	**Asp56**	**Arg403**	**-28.59**	**3.02**	**--**	H	Asp56	Tyr505	-3.1	2.64	b-	H	Asp56	His505	-2	2.84	b-
**IH**	**Asp56**	**Lys417**	**-23.26**	**2.73**	**--**	H	Lys59	Gln498	-3.3	2.91	b-	H	Asp57	Tyr501	-1.6	2.91	b-
H	Asp56	Tyr505	-1.9	2.69	b-	H	Tyr60	Gly446	-0.5	2.87	b	H	Asn58	Ser496	-2.3	2.66	--
H	Asn58	Arg403	-0.6	3.03	--	H	Lys66	Gln498	-9	2.74	--	H	Lys59	Arg498	-5.7	2.84	b-
H	Lys59	Tyr449	-3.3	2.68	b-	H	Arg105	Gly446	-5.5	3.07	b	H	Pro101	Tyr489	-2.6	2.84	b-
H	Tyr60	Ser494	-2.8	2.83	b	H	Arg105	Gln493	-1.3	2.95	b-	H	Arg105	Ser494	-3.9	2.92	bb
H	Lys66	Gly446	-7.9	2.79	b	H	Arg105	Ser494	-5.8	2.83	bb	H	Arg107	Asn450	-11.3	3.01	-*
H	Lys66	Tyr449	-1	2.92	--	A	Tyr106	Phe490	0	3.83	--	H	Arg107	Ser494	-1.1	3.16	b-
H	Lys66	Gln498	-6.7	2.72	--	H	Arg107	Asn450	-6.2	2.79	b						
H	Arg105	Gln493	-3.9	2.82	b-	**IH**	**Arg109**	**Glu484**	**-27.45**	**2.75**	--						
H	Arg105	Ser494	-4.4	2.78	bb												
H	Arg107	Asn450	-11.2	2.96	-*												
H	Arg107	Ser494	-1.2	3.16	b-												
**IH**	**Arg109**	**Glu484**	**-33.86**	**3**	**--**												
A	Tyr111	Tyr489	-0.7	4.67	--												

Table S2. Comparative interface of Etesevimab with RBD of wild type and Omicron Variant of SARS-CoV-2.

Etesevimab-RBD(WT)						Etesevimab-RBD(Omicron)					
Type	Etesevimab	RBD	Energy	Dist	BB	Type	Etesevimab	RBD	Energy	Dist	BB
H	Gly26	Asn487	-2.8	2.75	b-	H	Gly26	Asn477	-0.8	3.45	bb
H	Thr28	Ala475	-4.1	3.03	bb	H	Thr28	Ala475	-4.3	2.95	bb
H	Ser31	Tyr473	-3.1	2.69	b-	H	Thr28	Asn477	-0.7	3.32	--
H	Asn32	Ala475	-3.4	2.71	b	H	Ser31	Lys458	-4.1	2.83	--
A	Tyr33	Tyr421	-0.5	4.37	--	H	Ser31	Tyr473	-3.2	2.77	b-
H	Tyr33	Leu455	-3.9	2.63	b	H	Asn32	Ala475	-2.5	2.8	b
H	Gly54	Asn460	-0.6	3.18	b-	H	Tyr33	Leu455	-3.3	2.64	b
H	Ser56	Asp420	-0.9	2.59	--	H	Tyr52	Asn417	-3.2	2.69	--
H	Ser56	Asn460	-1.6	3.13	--	H	Gly54	Tyr421	-1.1	3.19	b-
H	Tyr92	Arg403	-9.2	2.88	b-	H	Ser56	Asp420	-1.7	2.65	--
H	Arg97	Asn487	-8	2.8	--	H	Ser56	Asn460	-2.2	2.89	--
H	Pro100	Lys417	-2.4	3.45	b-	H	Tyr92	Arg403	-8.7	2.75	b-
AH	Tyr102	Gln493	-3.6	3.43	*-	H	Tyr92	Gly502	-0.6	2.83	b
IH	Asp104	Lys417	-29.03	2.77	--	H	Arg97	Asn487	-7.1	2.88	--
						A	Tyr102	Lys493	-1	3.78	--

Table S3. CT-p59 escape estimation of the SARS-CoV-2 Spike mutant based on RBD mutations. Sorted according to Increase in resistance

Mutation	Resistance	Stability	Mutation	Resistance	Stability	Mutation	Resistance	Stability	Mutation	Resistance	Stability	Mutation	Resistance	Stability
Q493R	-8.88519	-0.5254133	N450K	-0.923	1.935703	N501H	0.592214	0.745792	R403Q	2.023199	2.637634	Q498K	4.179498	1.123557
Q493K	-7.83001	0.0094737	G446A	-0.92064	-0.08403	L455W	0.6535	2.622701	G485A	2.080001	-0.01907	Y449N	4.357169	3.490526
G446R	-6.29881	-0.0624447	E484D	-0.9158	0.838712	N501I	0.704258	0.184223	F456V	2.108419	1.768338	G485D	4.373046	0.700537
E406K	-4.81043	0.7437177	F456Y	-0.8587	0.891599	S494A	0.815601	0.675404	F456I	2.175259	1.55645	K417I	4.388513	1.629927
L455F	-4.44641	1.4308489	K417R	-0.80628	1.101501	L452S	0.875952	2.542673	K417T	2.199044	0.997066	Y453S	4.38936	3.026518
G446W	-3.60547	0.0486618	K417M	-0.62227	0.830266	G446S	0.878392	0.37837	F486L	2.227907	1.145301	R403S	4.423879	3.191042
Y449F	-3.52475	0.6005214	L492R	-0.59931	1.453927	Y489F	0.890462	0.529565	L452F	2.331136	1.833241	F486C	4.487392	2.107713
T478R	-3.36913	0.2737585	N501S	-0.58505	1.293792	K417Q	0.898777	1.0364	Y489H	2.332474	2.161817	R403L	4.709331	1.442575
E406D	-3.30982	1.358987	T478S	-0.53571	1.067641	Y449C	1.069321	2.632069	Y449D	2.370394	2.844526	R403I	4.839519	1.845699
L455M	-3.29173	1.7988501	Q498E	-0.3777	0.365114	E406Q	1.152319	1.31042	F456C	2.426827	2.75E+00	Y489C	4.921053	2.593694
N450Y	-3.24682	0.291158	Q493P	-0.3447	1.244743	S494T	1.176949	0.697646	Y505C	2.494709	1.42E+00	R403N	4.977428	3.305924
N501K	-3.11237	0.6024875	G446V	-0.24384	0.099365	R403W	1.226832	1.18292	F456S	2.514645	3.14E+00	R403G	5.507726	4.008353
Q493E	-3.0793	0.8971323	T478N	-0.23782	1.560161	F490I	1.294081	1.083041	Y505D	2.516695	2.04E+00	S494Y	5.530627	1.299329
Q493H	-2.87881	0.1845991	T478A	-0.18589	1.009268	G496W	1.302711	-0.70906	R403H	2.568383	1.667156	Y489S	5.596713	3.023975
Q493L	-2.83822	-1.3574476	L492F	-0.16134	0.765632	Y449S	1.322512	3.062585	Q498H	2.607292	0.280158	L452R	5.839492	1.934971
E484Q	-2.61392	0.5448936	K417N	-0.14619	1.228338	L492P	1.328979	2.152434	F490C	2.688395	2.326859	S494F	5.888696	2.056417
G446C	-2.26435	0.0551497	T478M	-0.13471	0.512994	N501Y	1.352706	0.297168	Q498L	2.738129	-0.67852	Y453D	6.10473	3.343695
E484A	-2.25966	1.2311797	L492M	-0.12151	1.424922	L455V	1.362644	1.185072	R403K	2.817791	1.778837	R403C	6.112456	2.856114
Y505H	-2.16697	1.1565951	L452I	-0.08556	0.63534	Q498R	1.367365	0.337209	L455S	2.832269	2.716076	R403T	6.135465	2.751813
L455R	-2.07911	1.5919169	E406V	-0.04688	0.979402	F490V	1.393135	1.256139	Q498P	2.854166	0.984963	R403P	6.634779	3.021981
Y449H	-1.99905	2.2085069	L492Q	-0.03838	2.009886	L492S	1.428475	2.490384	L455P	2.864947	2.781866	S494P	7.35467	2.191185
L455Q	-1.98447	2.5583496	L492H	0.022604	2.037572	G485V	1.456716	-0.12332	Y505S	2.885402	1.634067	G496R	7.72031	2.066436
E406A	-1.96944	1.2449301	T478P	0.063269	1.267265	G485S	1.492031	0.152656	G485E	3.057565	0.738954	S494L	7.934915	2.161342
T478K	-1.90104	1.0181617	G496A	0.0766	0.020034	F456L	1.516549	1.36945	Y453F	3.061283	0.654496	S494W	10.74292	1.315201
N450I	-1.87297	-0.5536203	L452H	0.083676	1.811109	L452P	1.538426	1.976772	Y495D	3.155566	2.869742			
G496C	-1.71698	-0.4776074	E406G	0.090598	2.402872	N501D	1.579818	1.201622	N501T	3.2076	0.715494			
L455I	-1.64026	1.1171491	S494C	0.136687	0.726328	Y453H	1.60313	2.491554	E484G	3.228465	2.204273			
L452V	-1.63348	1.1078742	E484V	0.167651	0.200829	F490L	1.634798	1.363714	Y495C	3.295346	2.813468			
G496S	-1.46399	0.1660893	Y495F	0.181118	0.634656	G485C	1.691806	0.054197	Y453N	3.607276	2.734868			
G485R	-1.40937	-0.2412279	L452Q	0.278192	2.08661	G496E	1.717046	0.654343	Y495S	3.636081	3.11484			
G446E	-1.29602	0.3555745	Y505F	0.295745	0.634415	K417E	1.722805	0.788634	Y495N	3.66515	2.870907			
N450H	-1.28691	0.8780861	F486Y	0.328987	1.219863	G496D	1.7766	0.810518	Y489N	3.74501	2.778746			
Y495H	-1.2151	1.9124419	N450D	0.347901	0.427112	F490S	1.815928	2.343692	R403M	3.832625	1.908511			
L492W	-1.19704	1.6899465	T478I	0.478888	0.283233	G485W	1.912471	0.172682	Y489D	3.849109	2.699395			
E484K	-1.12329	0.3675459	L492V	0.52297	1.137516	G496V	1.924842	-0.4901	F486S	3.908013	2.175798			
L452W	-1.09297	1.5996092	N450T	0.553652	0.033344	Y505N	1.925792	1.438027	Y453C	3.959365	2.826286			
F490Y	-1.01345	0.9176806	L492I	0.582787	0.593125	L452M	1.932548	1.280845	F486I	4.00632	0.896382			
G446D	-0.96633	0.3388248	N450S	0.584421	0.41683	L455H	1.952039	1.818497	F486V	4.022485	1.088906			

Table S4: CDR diversification of CT-59 against Delta strains.

CDRh1		
mutation	dAffinity	dStability
2:S30W	-1.44258	-0.1769
2:S32Y	-7.4292	-0.31001
2:S32W	-5.80866	-0.01821
2:S32R	-5.2869	-0.38031
2:S32L	-4.40036	-0.54318
2:G33H	-9.50115	-0.42554
2:G33W	-8.45517	-0.80317
2:G33Y	-8.19838	-0.8781
2:G33Q	-7.2651	-0.3824
2:G33T	-6.84999	-0.38801
2:G33M	-6.67402	-0.8148
2:G33R	-6.43444	-0.78488
2:G33I	-5.8307	-1.348
2:G33F	-5.65356	-0.96525
2:G33E	-5.17292	-0.143
2:G33P	-4.58133	-0.68409
2:G33C	-4.45403	-0.99331
2:G33V	-4.26949	-0.81202
2:G33S	-4.22141	-0.73895
2:G33N	-3.79675	-0.37614
2:G33A	-2.84884	-0.80233
2:G33D	-2.65823	-0.05091
2:G33L	-2.4673	-0.78355
2:V34I	-2.86352	-0.36124
2:G35N	-2.76741	-0.42578
2:G35R	-2.15019	-0.51103
2:G35M	-1.8882	-1.16925
2:G35Q	-1.73994	-0.71465

CDRh2		
mutation	dAffinity	dStability
2:D54W	-4.85	-0.95551
2:D54R	-4.57476	-0.83694
2:D54F	-2.75994	-0.74007
2:D54I	-1.72675	-1.31817
2:D54L	-1.24058	-0.86676
2:D54M	-1.15242	-0.54795
2:D54Y	-1.11307	-0.66204
2:D56I	-2.09833	-0.55836
2:D56K	-1.8478	-0.12091
2:D57I	-2.19687	-0.54121
2:D57Y	-1.57515	-0.15905
2:N58W	-13.9957	-2.25274
2:N58Y	-11.0665	-1.26811
2:N58R	-8.95788	-1.03486
2:N58F	-8.40805	-2.23546
2:N58K	-7.09599	-0.20386
2:N58M	-5.95307	-0.46463
2:N58H	-5.79993	-0.15717
2:N58Q	-5.03937	-0.06805
2:N58V	-3.64637	-0.89936
2:N58I	-2.83081	-0.64924
2:N58P	-2.7702	-0.22522
2:N58L	-1.41571	-0.44507
2:K59W	-5.99913	-0.65031
2:K59Y	-4.928	-0.18931
2:K59F	-3.82176	-0.45436
2:K59I	-1.58948	-0.21665
2:K59L	-1.24833	-0.29522

CDRh3		
mutation	dAffinity	dStability
2:Y106I	-6.70378	-0.65763
2:Y106V	-2.89638	-0.12121
2:Y106L	-2.38204	-0.30364
2:R107D	-6.90378	-0.75763
2:N108R	-3.67322	-0.44445
2:N108L	-2.4069	-0.8843
2:N108Q	-1.08204	-0.61456
2:Y110W	-3.2768	0.006935

CDRL2		
mutation	dAffinity	dStability
1:D51Y	-7.16424	-1.1083
1:D51W	-6.65637	-0.937
1:D51F	-5.69976	-1.21293
1:D51T	-2.27342	-0.22977
1:N52R	-1.57768	-0.44508
1:N52Y	-1.13739	-0.05544
1:N52F	-1.09776	-0.36274
1:N52L	-1.02759	-0.59508

CDRL3		
mutation	dAffinity	dStability
1:S97W	-6.10794	-0.57483
1:S97F	-3.35727	-1.2129
1:S97R	-2.29344	-0.48253
1:S97M	-1.34158	-0.67491

The dStability and dAffinity terms: Change in the relative stability of the Ab-Ag complex upon mutation. Given values is a difference between values for the wild-type amino acid and mutant amino acid. Negative values represent better stability and low resistance.

Table S5: Mutations carried in the CDRs of CoVAb1-9

	CDRL1	CDRL2	CDRL3	CDRH1	CDRH2	CDRH3
**CT-p59**	SSNIGNNY	DNN	GTWDSSLSAGV	GFSLSTSGVG	IDWDDNK	ARIPGFLRYRNRYYYYGMDV
**CoVAb1**	SSNIGNNY	DNN	GTWDSSLWAGV	GFSLSTSHVG	IDWDDYW	ARIPGFLRYRNRYYYYGMDV
**CoVAb2**	SSNIGNNY	WNN	GTWDSSLWAGV	GFSLSTSHVG	IDWDWYL	ARIPGFLRYRNRYYYYGMDV
**CoVAb3**	SSNIGNNY	FNN	GTWDSSLWAGV	GFSLSTSHVG	IDWDWYI	ARIPGFLRYRNRYYYYGMDV
**CoVAb4**	SSNIGNNY	FNN	GTWDSSLWAGV	GFSLSTSTVG	IDWDWNK	ARIPGFLRYRNRYYYYGMDV
**CoVAb5**	SSNIGNNY	FNN	GTWDSSLWAGV	GFSLSTSTVG	IDWDWNF	ARIPGFLRYRNRYYYYGMDV
**CoVAb6**	SSNIGNNY	DNN	GTWDSSLSAGV	GFSLSTDGVG	IDWDDWK	ARIPGFLRYDNRYYYYGMDV
**CoVAb7**	SSNIGNNY	DNN	GTWDSSLSAGV	GFSLSTDWVG	IDWDDWK	ARIPGFLRWDNRYYYYGMDV
**CoVAb8**	SSNIGNNY	YNN	GTWDSSLFAGV	GFSLSTSGVG	IDWDDWK	ARIPGFLRYRNRYYYYGMDV
**CoVAb9**	SSNIGNNY	DNN	GTWDSSLFAGV	GFSLSTSWVG	IDWDDYK	ARIPGFLRYRNRYYYYGMDV

Table S6: CDR diversification of CT-59 against wild-type strain

CDRH2				CDRH1		
**mutation**	**dAffinity**	**dStability**		**mutation**	**dAffinity**	**dStability**
2:D56F	-3.80917	0.085522		2:S30W	-2.08697	0.001316
2:D57W	-5.77134	-1.61901		2:S32Y	-5.52693	-0.52228
2:D57F	-4.22257	-1.74427		2:S32F	-2.77969	-0.36004
2:D57Q	-3.47727	-0.47579		2:G33R	-4.76436	0.003217
2:D57I	-3.06603	-1.57917		2:G33S	-2.8429	-0.24206
2:D57H	-2.29684	-0.56927		2:G33F	-2.80465	-0.57569
2:D57M	-2.2699	-0.86733		2:G33Y	-2.67478	-0.55067
2:D57Y	-1.90874	-1.49851		2:G33Q	-2.34141	-0.33774
2:D57R	-1.44098	-0.62987		2:G33C	-1.9832	-0.37656
2:K59W	-2.60814	-0.01267		2:G33V	-1.81624	-1.08265
				2:G33A	-1.74338	-0.25091
				2:G33E	-1.32832	0.035629

CDRL3				CDRL2		
**mutation**	**dAffinity**	**dStability**		**mutation**	**dAffinity**	**dStability**
1:S97Y	-2.25057	-0.08481		1:D51Y	-3.90872	-0.82596
1:S97M	-1.68604	-0.07702		1:D51W	-3.70816	-0.85037
1:S97F	-1.6508	-0.68135		1:D51F	-3.16845	-0.79193
				1:N52R	-1.57255	-0.38875

The dStability and dAffinity terms: Change in the relative stability of the Ab-Ag complex upon mutation. Given values is a difference between values for the wild-type amino acid and mutant amino acid. Negative values represent better stability and low resistance.

Table S7. Interface of CoVAb6 and CoVAb7 with RBD^{Omicron} of SARS-CoV-2.

CoVAb6-Delta						CoVAb7-Delta					
**Type**	**CoVAb6**	**RBD**	**Energy**	**Dist**	**BB**	**Type**	**CoVAb7**	**RBD**	**Energy**	**Dist**	**BB**
**IH**	**Asp32**	**Lys417**	**-14.98**	**2.9**	--	**IH**	**Asp32**	**Lys417**	**-15.08**	**3.05**	--
H	Tyr33	Glu484	-1.8	2.76	b-	H	Tyr33	Glu484	-2.3	2.63	b-
H	Asp51	Phe486	-1	2.84	b-	H	Asp51	Gly485	-1	3.11	b-
**IH**	**Asp56**	**Arg403**	**-28.22**	**2.94**	--	**IH**	**Asp56**	**Arg403**	**-21.06**	**3.12**	--
H	Asp56	Tyr453	-0.5	3.49	--	H	Asp56	Tyr453	-1.4	3.04	--
H	Trp58	Asn501	-1.2	3.2	--	H	Arg105	Gln493	-5.4	3	--
H	Arg105	Gln493	-2.2	3.06	**	H	Arg105	Ser494	-6.2	2.81	*b
H	Arg105	Ser494	-10.5	2.94	*b	H	Trp106	Glu484	-0.5	3.38	--
A	Tyr106	Phe490	0	3.61	--	A	Trp106	Phe490	0	3.55	--
**IH**	**Asp107**	**Arg452**	**-33.6**	**2.98**	--	**IH**	**Asp107**	**Arg452**	**-34.85**	**2.97**	--
H	Asp107	Ser494	-1.7	3.06	b-	H	Asp107	Ser494	-1.2	2.92	b-
A	Asn108	Tyr449	-0.8	3.54	--	A	Asn108	Tyr449	-0.8	3.64	--
**IH**	**Arg109**	**Glu484**	**-32.92**	**2.99**	--	**IH**	**Arg109**	**Glu484**	**-37.92**	**2.85**	**--**

Table S8. Interface of Etsimic1 and Etsimic2 with RBD^{Delta} of SARS-CoV-2.

Etesimic1-Omicron						Etesimic2-Omicron					
Type	Etesimic1	RBD	Energy	Dist	BB	Type	Etesimic2	RBD	Energy	Dist	BB
H	Asp26	Asn477	-5.5	2.77	bb	H	Asp26	Asn477	-5.6	2.76	bb
**IH**	**Asp26**	**Lys478**	**-19.04**	**3.11**	**--**	**IH**	**Asp26**	**Lys478**	**-10.75**	**3.13**	**--**
H	Asp26	Asn487	-6.4	2.77	--	H	Asp26	Asn487	-4.4	2.72	--
H	Ser28	Gly502	-3.8	2.88	bb	H	Phe27	Asn477	-0.9	2.9	b-
H	Thr28	Ala475	-4	2.85	bb	H	Ser28	Thr500	-2	2.88	b
H	Thr28	Asn477	-2.4	2.68	--	H	Ser28	Gly502	-2.3	2.99	bb
**IH**	**Glu31**	**Arg498**	**-20.06**	**3.04**	**--**	H	Thr28	Ala475	-4.6	2.8	bb
H	Ser31	Tyr473	-3.3	2.79	b-	H	Thr28	Asn477	-1.4	2.69	--
H	Asn32	Ala475	-2.7	2.88	b	H	Glu31	Ser496	-2	2.8	--
**I**	**Glu32**	**Arg403**	**-2.79**	**3.29**	--	**IH**	**Glu31**	**Arg498**	**-25.55**	**2.78**	**--**
H	Glu32	His505	-2.6	2.84	--	H	Ser31	Lys458	-0.5	3.44	--
H	Tyr33	Leu455	-3.4	2.67	b	H	Ser31	Tyr473	-3.2	2.63	b-
H	Tyr52	Asn417	-3.2	2.69	--	H	Asn32	Ala475	-2.3	3.2	b
H	Ser56	Asp420	-1.3	2.6	--	**IH**	**Glu32**	**Arg403**	**-19.21**	**3.25**	**--**
H	Tyr92	Arg403	-4.7	2.77	b-	H	Glu32	His505	-2.7	2.77	--
H	Thr94	Arg408	-0.6	3.08	b-	H	Tyr33	Leu455	-3.3	2.69	b
H	Arg97	Asn487	-5.3	2.73	--	H	Ser56	Asp420	-1.9	2.78	--
**IH**	**Glu102**	**Lys493**	**-18.33**	**2.71**	--	H	Tyr92	Arg403	-1.5	2.84	b-
						H	Arg97	Asn487	-7.9	2.72	--
						H	Met102	Lys493	-0.5	3.48	--
